# Address to the Graduating Class of the Baltimore College of Dental Surgery, Previously to Conferring the Degrees, at the Ninth Annual Commencement, Held March 1st, 1849.

**Published:** 1849-01

**Authors:** Eleazar Parmly

**Affiliations:** Provost of the Institution.


### ARTICLE V.

*Address to the Graduating Class of the Baltimore College of Dental Surgery, previously to conferring the Degrees, at the Ninth Annual Commencement, held March* 1*st*, 1849. By Eleazar Parmly, M. D., D. D. S., Provost of the Institution.

My young friends—Graduates of the Baltimore College of Dental Surgery—it has been made my very pleasing duty this day to act as the duly constituted organ of the College, in presenting its diploma to the graduating class. If it affords you as much satisfaction to *receive* as it does me to *confer* this honor in the name of the Faculty of the College, the present is an occasion not soon to be forgotten.

The present ceremony will ever be regarded by each of you as constituting a memorable era in life, inasmuch as it terminates the period of preliminary preparation, and commences the period of professional practice; the former may be regarded as the *minority*, the latter the *majority*, of professional life; and this fact alone carries with it a weight of individual responsibility, from which I trust you will not be anxious to escape.

Whatever may have been your dependence in days that are passed, on parents, friends or teachers, the time has now come when you must learn the lesson of self-reliance—and if you are fully aware how much this is dependent on self-respect, you are in some measure prepared to act for yourselves. There are two aspects in which this self-respect, so essential to professional distinction, should be regarded: the one relates to personal morals, the other to professional qualifications. It may be safely averred that in no calling in civilized society are moral character, honorable principles and strict integrity more imperatively required, in order to secure that public confidence which alone can secure success, than in that to which the degree about to be conferred is your honorable and accredited introduction. It will probably be your fortune, as it has been mine, to hear the bitter lamentations of certain worthless aspirants after public favor in our profession, whose utter disappointment is the natural result of immoral practices and immoral indulgences. Among these, I would particularly allude to one, and most emphatically urge it upon your consideration, and most earnestly caution you against it, because it is more likely than any other to involve both your social and professional prospects in inevitable ruin and destruction; and that one is, indulgence in early life in that practice which may eventually lead to confirmed dissipation. Men of otherwise reputable attainments come wholly short of professional success for want of a good moral character. On this point there is little danger that any of your advisers should say too much; but I must submit the whole subject to your sober reflection and individual application, suggesting for your serious inquiry whether, of all sublunary goods, good morals are not the best. The merely intellectual man may be learned and great, but the virtuous and the honorable man is alone wise and good.

Next to excellence of moral character, your self-respect, which is the only sure foundation for the respect of others, must be built and established on professional competence and merit. Of this competence, although the diploma of this College may not be an unerring indication in every instance, it is unquestionably the surest pledge of merit which the public can receive in the present state of dental education in our country. Of this fact, neither the Faculty of this College nor he who addresses you are the only believers—our fellow citizens generally, in many of the states, have already manifested their respect for this institution and its diploma, by confiding fearlessly in those who have hitherto received it. Even in foreign lands remote, its agency will be found to exert a powerful influence in favor of its possessor, opening for him at once the avenue to success.

But, as we have already more than intimated, permanent success must depend upon positive professional merit, without which no honorable man can either respect himself, or command the respect of others.

You scarcely need be told that those preliminary attainments which procure for you this day the honorable degree of Doctor of Dental Surgery, cannot be regarded as anything more than the acquirements of an accomplished apprentice compared with the skill of the experienced and finished master. But to that superior master-workmanship let it be your high ambition to attain, in due process of time, by industry, observation and experience. Set your mark high, and strive to reach it, always remembering that it is more honorable to fall short of a noble end, than to obtain a mean one.

Your first aim and object in professional life should be to be useful, and in being so to your patrons, you will be so to yourselves, to the community in which you live, to your country, and to your generation. In order to become useful as dentists, you most deal in matters of fact, and not in matters of fancy—in common sense, and not in idle speculation—in that which leads to good practical results, instead of failure and disappointment—in doing, instead of boasting what you can do; the dentist of deeds and not of words will always be chosen; and the life of him who faithfully discharges his duty will be a life of labor. The white-gloved and soft-handed dentist of George IV, in speaking of Waite, the best operator then in England, spoke contemptuously of his hard hand. It is better, my friends, to have hard hands, made so by professional labor, than to have nothing better to relieve your patients than soft words and softer pretensions; one proves that you are not ashamed of labor nor of your profession—the other proves that you are fit for neither the one nor the other. There may be such as will speak slightly of your profession and of the duties you have to perform, but you can always point to those in the highest, as well as those in the lowest, walks of life, who have been the most useful, the most beloved, and who have conferred the greatest benefits on their fellow-creatures, who in early life learned lessons of industry, and learned, too, to respect labor; and however humble their occupations they have felt only equal to it, never above it. You will find the greatest benefit from consulting persons older than yourselves in the profession. There are perhaps an equal number of those who underrate as those who overrate their abilities, and both of those classes lose the benefit of the knowledge and experience of their seniors. The first, from want of confidence in themselves, or from diffidence, are afraid to approach those who have been long in practice. The second class never approach them, because they believe they have nothing to learn; their self-pride is such that they would feel humbled in either asking or taking advice. Beware, my young friends, of arrogance and pride—they ruin the success and reputation of more dentists in the estimation of the public, and confer fewer benefits, than anything I know of except imposture and laziness. The confidence and name gained by generous acts will be of much higher value to you than money gained by mean ones. Meanness in underrating and underpricing your contemporaries should be no part of a professional man's character. It degrades instead of elevates any and every one who will do it. You can succeed, if you will, without such unmanly and ignoble artifices. Perseverance and industry, with the knowledge you now possess, never has and never will fail of accomplishing all you desire.

I read, a short time ago, from a document written by a dentist of some twenty years' experience, that "dentistry is an art acquired by observation and practice—practice is nearly every thing—theory very little." There may be others who would express the same opinion; but when they do so, they show themselves wholly unacquainted with, and ignorant of, the best and true sources of professional knowledge, and an entire indifference to the value of correct theory, from which must result all correct practice. This remark should be exactly reversed with the young practitioner: theory is nearly every thing—practice, or the mere manipulation, is comparatively very little, as you must all have experienced that it is very easy to learn to do a thing after you have been carefully and thoroughly instructed in the best way or mode of doing it. I affirm, and do so without the fear of contradiction, that the true surgical and mechanical principles or science of our art, your acquaintance with, and your ability to perform safely, the delicate and beautiful operations exhibited by graduates of this institution, never was and never will be attained by any one having only the advantage of observation from his own practice. It is now an easy matter to make a lightning rod or construct telegraphic wires; but it required the ablest minds to originate and entertain the theory of electric transmission; and we might, with the same justice and propriety, say that the making of the wires is nearly every thing, and the mind which conceived, and the theory which established, this truly wonderful communication, very little. It is of much greater consequence, my young friends, to yourselves, to your profession, and infinitely greater to your patients, for you to know when and how to perform an operation so as to avoid risk or danger, than merely to be able mechanically to do it well, and for the want of such knowledge hazard the health, vitality, or may be the entire loss of the teeth upon which you have operated. In this way there is, at this very period, a greater amount of injury than good done to teeth, and that, too, by the recklessness of persons who submit themselves, without knowledge or proper precaution, to dentists more reckless than themselves.

I trust, in your professional practice, you will never forget that you are graduates of the Baltimore College—the first institution of the kind established in the world; and we have great confidence and strong faith in your efforts to make it known, and regard it not only as first in origin, but first in the character and qualifications of its graduates. And may you become as distinguished in your calling for the superiority of your professional tact and attainments over that of any other system of instruction, and gain as much credit for yourselves, and reflect as much honor on your profession, your instructors and upon this institution, in contending for truth and correct professional practice against error, imposture and presumption, as our noble youths, the graduates of the Academy of West Point, have for themselves, their country and their institution, in their late triumphant conflicts with their enemies on the battle grounds of Mexico. It is with no small degree of pride and pleasure I speak of a sister institution, established on the same basis, in Cincinnati, Ohio, one of the fields of my early professional efforts. We congratulate its professors and teachers in their laudable designs and philanthropic labors; we commend their enterprise, and most cordially extend to them our paternal regards and ardent wishes for their abiding welfare and prosperity, and most earnestly do we hope they will not fail to attain the highest object vouchsafed to them by their professional zeal and praiseworthy ambition. And what more shall I say, after all the valuable instruction which has fallen from your teachers previous to this occasion, than now to invite you to receive the diploma of the Baltimore College of Dental Surgery, as a token of your creditable attainments in dental science and art. May it be to each of you a sure passport to fame and fortune; but, above all, may your own professional merit soon place you, one and all, above all necessity to any passports to the hearts of your countrymen, or, if occasion may require, the favor of strangers in a foreign land.

---

### *New Remedy for Tooth-Ache.*

MR. JAMES BEATSON, of Airdrie, says: Gum copal, when dissolved in chloroform, forms an excellent compound for stuffing the holes of decayed teeth. I have used it very frequently within the last two weeks, and the benefit which my patients have derived from it has been truly astonishing. The application is simple and easy. I clean out the hole, and moisten a little cotton with the solution; I introduce this into the decayed part, and in every instance the relief has been almost instantaneous. The chloroform removes the pain, and the gum copal resists the action of the saliva, and, as the application is so agreeable, those who may labor under this dreadful malady would do well to make a trial of it.—*Medical Times.*

